# Supplementary figures and images for: Higher red cell distribution width (RDW) is associated with increased all-cause and cardiovascular mortality in patients with breast cancer: A retrospective analysis of NHANES data (1999–2018)
Source: PLoS One. 2025 Jul 28;20(7):e0328680. doi: 10.1371/journal.pone.0328680 (PMC12303287; doi:10.1371/journal.pone.0328680)

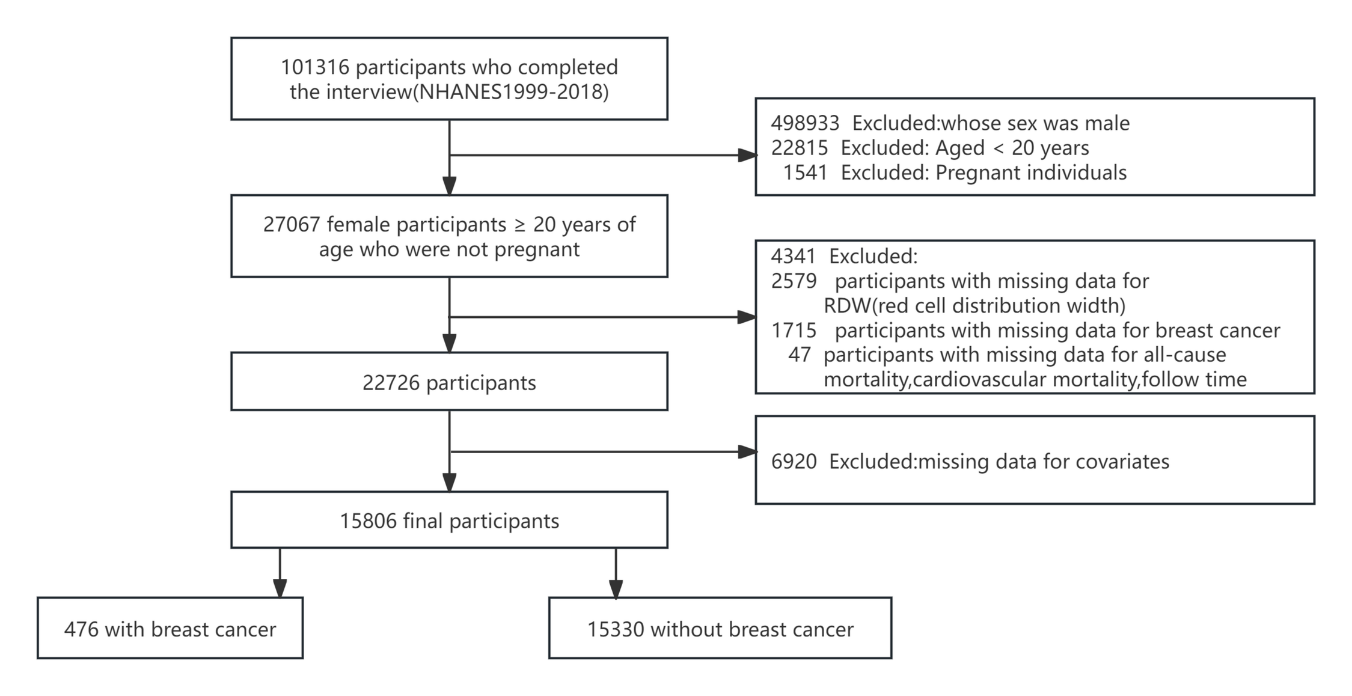


**Figure.S1 The ﬂow chart of the study.**

Supplement: S1 Fig — (DOCX) [file pone.0328680.s001.docx]
