# Supplementary material for: Higher red cell distribution width (RDW) is associated with increased all-cause and cardiovascular mortality in patients with breast cancer: A retrospective analysis of NHANES data (1999–2018)
Source: PLoS One. 2025 Jul 28;20(7):e0328680. doi: 10.1371/journal.pone.0328680 (PMC12303287; doi:10.1371/journal.pone.0328680)
Supplement: S1 Table — (DOCX) [file pone.0328680.s002.docx]

**Table S1. General characteristics of the study population according to breast cancer.**

| **Characteristic** | **Total** | **Without**  **Breast cancer** | **With**  **Breast cancer** | **p** |
| --- | --- | --- | --- | --- |
| Sample size, N | 15806 | 15330 | 476 |  |
| RDW (%) | 13.2 ± 1.5 | 13.2 ± 1.5 | 13.4 ± 1.2 | 0.018 |
| RDW group, n (%) |  |  |  | < 0.001 |
| Low RDW(<12.68%) | 6513 (41.2) | 6376 (41.6) | 137 (28.8) |  |
| High RDW(≥12.68%) | 9293 (58.8) | 8954 (58.4) | 339 (71.2) |  |
| Age(years) | 49.2 ± 17.7 | 48.7 ± 17.5 | 67.7 ± 11.6 | < 0.001 |
| Race, n (%) |  |  |  | < 0.001 |
| Non-Hispanic White | 2831 (17.9) | 2790 (18.2) | 41 (8.6) |  |
| Non-Hispanic Black | 1357 ( 8.6) | 1332 (8.7) | 25 (5.3) |  |
| Mexican American | 7141 (45.2) | 6832 (44.6) | 309 (64.9) |  |
| Other | 4477 (28.3) | 4376 (28.5) | 101 (21.2) |  |
| Education, n (%) |  |  |  | 0.593 |
| blow high school | 3987 (25.2) | 3875 (25.3) | 112 (23.5) |  |
| high school | 3527 (22.3) | 3423 (22.3) | 104 (21.8) |  |
| above high school | 8292 (52.5) | 8032 (52.4) | 260 (54.6) |  |
| Marital, n (%) |  |  |  | < 0.001 |
| Married | 8648 (54.7) | 8405 (54.8) | 243 (51.1) |  |
| Living alone | 4501 (28.5) | 4293 (28) | 208 (43.7) |  |
| Never married | 2657 (16.8) | 2632 (17.2) | 25 (5.3) |  |
| PIR, n (%) |  |  |  | < 0.001 |
| Low | 5039 (31.9) | 4925 (32.1) | 114 (23.9) |  |
| Medium | 5966 (37.7) | 5769 (37.6) | 197 (41.4) |  |
| High | 4801 (30.4) | 4636 (30.2) | 165 (34.7) |  |
| BMI (kg/m^2^) | 29.4 ± 7.4 | 29.4 ± 7.5 | 29.0 ± 6.9 | 0.204 |
| Hypertension, n (%) |  |  |  | < 0.001 |
| No | 9225 (58.4) | 9065 (59.1) | 160 (33.6) |  |
| Yes | 6581 (41.6) | 6265 (40.9) | 316 (66.4) |  |
| Heart failure, n (%) |  |  |  | < 0.001 |
| No | 15427 (97.6) | 14979 (97.7) | 448 (94.1) |  |
| Yes | 379 ( 2.4) | 351 (2.3) | 28 (5.9) |  |
| Cardiovascular disease, n(%) |  |  |  | < 0.001 |
| No | 15426 (97.6) | 14977 (97.7) | 449 (94.3) |  |
| Yes | 380 ( 2.4) | 353 (2.3) | 27 (5.7) |  |
| Angina, n (%) |  |  |  | < 0.001 |
| No | 15433 (97.6) | 14984 (97.7) | 449 (94.3) |  |
| Yes | 373 ( 2.4) | 346 (2.3) | 27 (5.7) |  |
| Diabetes, n (%) |  |  |  | < 0.001 |
| No | 13451 (85.1) | 13097 (85.4) | 354 (74.4) |  |
| Yes | 2355 (14.9) | 2233 (14.6) | 122 (25.6) |  |
| Diabetes family history, n (%) |  |  |  | 0.018 |
| No | 8356 (52.9) | 8079 (52.7) | 277 (58.2) |  |
| Yes | 7450 (47.1) | 7251 (47.3) | 199 (41.8) |  |
| Hyperlipidemia, n(%) |  |  |  | < 0.001 |
| No | 9738 (61.6) | 9529 (62.2) | 209 (43.9) |  |
| Yes | 6068 (38.4) | 5801 (37.8) | 267 (56.1) |  |
| Hormone replacement  therapy, n (%) |  |  |  | 0.023 |
| No | 14261 (90.2) | 13846 (90.3) | 415 (87.2) |  |
| Yes | 1545 ( 9.8) | 1484 (9.7) | 61 (12.8) |  |
| Mcv, Mean ± SD | 88.8 ± 6.1 | 88.8 ± 6.1 | 90.3 ± 5.3 | < 0.001 |
| Total cholesterol(mg/dL) | 198.4 ± 41.0 | 198.2 ± 40.9 | 204.2 ± 42.3 | 0.001 |
| High density lipoprotein  (mg/dL) | 57.3 ± 16.1 | 57.2 ± 16.1 | 59.2 ± 16.8 | 0.009 |
| HbA1c (%) | 5.7 ± 1.1 | 5.7 ± 1.1 | 5.9 ± 0.9 | < 0.001 |
| Hemoglobin (mg/dL) | 13.4 ± 1.3 | 13.4 ± 1.3 | 13.4 ± 1.2 | 0.656 |
| Albumin, (mg/dL) | 4.2 ± 0.3 | 4.2 ± 0.3 | 4.1 ± 0.3 | 0.003 |
| Serum creatinine (mg/dL) | 0.7 (0.6, 0.8) | 0.7 (0.6, 0.8) | 0.8 (0.7, 1.0) | < 0.001 |
| eGFR, Mean ± SD | 95.6 ± 23.1 | 96.1 ± 22.9 | 78.0 ± 20.7 | < 0.001 |

PIR: Poverty income ratio; BMI: body mass index; CVD: cardiovascular disease; FHD: Family history of diabetes; TC: Total cholesterol; HDL: high-density lipoprotein cholesterol; TG: triglyceride; BUN: Blood urea nitrogen; SCR: serum creatinine; HbA1c: Glycosylated hemoglobin; Hb: hemoglobin; eGFR: estimated glomerular filtration rate; MCV, mean corpuscular volume; RDW, red cell distribution width;

Continuous variables are presented as the mean and 95% confidence interval, category variables are described as the percentage and 95% confidence interval.
