# Supplementary material for: Higher red cell distribution width (RDW) is associated with increased all-cause and cardiovascular mortality in patients with breast cancer: A retrospective analysis of NHANES data (1999–2018)
Source: PLoS One. 2025 Jul 28;20(7):e0328680. doi: 10.1371/journal.pone.0328680 (PMC12303287; doi:10.1371/journal.pone.0328680)
Supplement: Attachment — Submitted filename: Response to Reviewers .docx [file pone.0328680.s005.docx]

**Higher Red Cell Distribution Width (RDW) Associated with Increased Risk of All-Cause and Cardiovascular Mortality in Breast Cancer Individuals: A Retrospective Cohort Analysis from NHANES 1999-2018**

**[PONE-D-24-49688] - [EMID:8a3f1f606cb48a6f]**

Dear Editor and Reviewers.

On behalf of my co-authors, we greatly appreciate the careful review and comments from both you and the reviewers. We have carefully considered all the comments and have made the necessary revisions to improve the quality of our paper. Below, we provide a detailed response to each of the reviewers comments and describe the changes made to the manuscript. There are no conflicts of interest regarding this work. All authors have read the revised manuscript and approved its submission to the *PLOS ONE*. Please do not hesitate to contact us if we can be of any further assistance.

Thank you and best regards.

Yours Sincerely.

Jinmin Cao

E-mail: [caojinmin_cool@126.com](mailto:caojinmin_cool@126.com)

Department of Dermatology, Hunan Aerospace Hospital, Changsha, Hunan, China

**Editor**

**1、Response to Editor**

Dear Editor,

Thank you very much for your careful review of our manuscript and for raising these important queries. We truly appreciate the opportunity to address your concerns and improve the quality of our work.

We will carefully and thoroughly address each of your queries in a detailed response, providing the necessary clarifications and justifications to ensure the robustness and validity of our study.

Once again, thank you for your time and valuable feedback.

**Comment 1: Selection of Exposure Measurement: The manuscript does not indicate when RDW was measured relative to cancer diagnosis and subsequent mortality outcomes. Was RDW assessed at baseline only, or were repeated measurements used? Please clarify the timing and frequency of RDW assessment. Additionally, please provide justification for using RDW as a stable prognostic biomarker in this context, and whether its variability over time was considered.**

**Response 1.1：**

We sincerely appreciate the reviewer's insightful comments and constructive questions, which have helped us clarify key methodological aspects of our study. We have carefully addressed each point below to enhance the transparency and rigor of our manuscript.

In the National Health and Nutrition Examination Survey (NHANES), hematologic parameters (including RDW and MCV) were obtained concurrently with questionnaire data (such as the Medical Conditions Questionnaire [MCQ]) during the same examination visit. NHANES is a cross-sectional survey and generally does not reassess participants over time; thus, only baseline RDW values were analyzed. Mortality outcomes were determined via linkage to the National Death Index (NDI) up to December 2019, allowing for survival analysis despite the lack of follow-up laboratory data.

The original text at Line 121-124:

**Ascertainment of RDW**

Red cell distribution width (RDW) determination was performed based on the full blood count data, utilizing a Beckman Coulter MAXM automated hematology analyzer to measure the values.

*Revised Manuscript at Lines 121-125:*

**Ascertainment of RDW**

*In our study, RDW was measured only at baseline during the initial examination visit, and no repeated measurements were conducted over time. Red cell distribution width (RDW) determination was performed based on the full blood count data, utilizing a Beckman Coulter MAXM automated hematology analyzer to measure the values.*

**Comment 1.2: Additionally, please provide justification for using RDW as a stable prognostic biomarker in this context, and whether its variability over time was considered.**

**Response 1.2：**

We sincerely appreciate the reviewer's insightful question regarding the use of red cell distribution width (RDW) as a stable prognostic biomarker in our study. Our justification is supported by the following points:

A substantial body of research has highlighted the association between red blood cell distribution width (RDW) and mortality outcomes across diverse patient cohorts, such as surgical patients[1], individuals with type 2 diabetes[2], those suffering from foot ulcers[3], burn victims[4], and critically ill patients[5]. These associations highlight the prognostic potential of RDW and its clinical relevance.

Despite the potential for RDW to fluctuate over time or in response to various physiological conditions (such as: nutritional status, inflammation, or cancer therapy), its prognostic power in chronic conditions like diabetes and cardiovascular disease has been robustly validated. In our referenced literature, studies employing the NHANES database have investigated the association between red blood cell distribution width to albumin level and all-cause mortality among patients with diabetic retinopathy and within the general population[6,7]. The single-time-point measurement of RDW still predicts long-term outcomes, indicating that its risk stratification capability remains stable over time. This is consistent with the design and objectives of our study.

While RDW is a convenient single-time-point biomarker, we recognize that its prognostic accuracy may be affected by unmeasured temporal variations. This limitation is addressed in the discussion section. While RDW is a practical single-time-point biomarker, this study acknowledges that its prognostic utility could be influenced by unmeasured temporal variability. This limitation is addressed in the discussion section (*Line 356-359:*). Future research could consider incorporating repeated RDW measurements to investigate its time-dependent effects, as the reviewer has appropriately suggested.

*(Line 356-359:*

*Limitation---Thirdly, measuring RDW at a single time point may not capture temporal fluctuations that could influence mortality risk. Furthermore, the study population was derived from NHANES data, which may restrict the generalizability of the findings to other populations with disparate characteristics.)*

**Comment 2: Methodological consideration: Model Building and Covariate Selection**

**Your three multivariable Cox regression models progressively adjust for a wide range of variables, but the rationale for including specific covariates is not explained. In particular, variables such as HbA1c, HDL-C, and serum creatinine may function as mediators of the RDW–mortality relationship rather than confounders. Please explain the conceptual framework (e.g., directed acyclic graphs or clinical reasoning) guiding your variable selection, and clarify whether model assumptions (such as the proportional hazards assumption) were assessed and met.**

**Comment 2.1: Your three multivariable Cox regression models progressively adjust for a wide range of variables, but the rationale for including specific covariates is not explained.**

**Response 2.1：**

Thank you very much for your valuable suggestions, which have taught me how to use Directed Acyclic Graphs (DAGs) **(Figure 1)** to determine the covariates to include in our multivariable model. This approach has helped streamline the number of covariates and improve the overall robustness of our model.

The inclusion of covariates was determined using a multifaceted approach, considering (1) documented results from the literature[8–11], (2) univariate Cox regression analysis results **(Table 1)** with *p* < 0.05, and (3) the impact of covariates on the exposure-outcome association, with a threshold set at more than 10%**(Table 2)**. The variables included are detailed below: Age, Sex, Race, Martial status, PIR, BMI, Hypertensive, Cardiovascular disease, angina, heart failure, diabetes, family history of diabetes, Hyperlipidemia, HbA1c, Total cholesterol, High density lipoprotein, Albumin, Hemoglobin, MCV, Serum creatinine, eGFR.

**Figure 1.** Directed Acyclic Graph (DAG) of Causal Pathways Between Red Cell Distribution Width (RDW) and Breast Cancer-Related Factors

**Table 1. Univariate Analysis for the RDW of Breast cancer**

| **Characteristic** | **OR（95%CI）** | **p_value** |
| --- | --- | --- |
| Age,years | 1.07 (1.07~1.08) | <0.001 |
| Race,% |  |  |
| Non-Hispanic White | 1 |  |
| Non-Hispanic Black | 1.28 (0.77~2.11) | 0.339 |
| Mexican American | 3.08 (2.22~4.28) | <0.001 |
| Other | 1.57 (1.09~2.26) | 0.016 |
| Education levels, % |  |  |
| blow high school | 1 |  |
| high school | 1.05 (0.8~1.38) | 0.718 |
| above high school | 1.12 (0.89~1.4) | 0.323 |
| Marital, % |  |  |
| Married | 1 |  |
| Living alone | 1.68 (1.39~2.02) | <0.001 |
| Never married | 0.33 (0.22~0.5) | <0.001 |
| PIR |  |  |
| ≤1.3 | 1 |  |
| 1.3-3.5 | 1.48 (1.17~1.86) | 0.001 |
| >3.5 | 1.54 (1.21~1.96) | <0.001 |
| BMI,kg/m^2^ | 0.99 (0.98~1) | 0.204 |
| Hypertensive, n (%) |  |  |
| No | 1 |  |
| Yes | 2.86 (2.36~3.47) | <0.001 |
| Heart failure, n (%) |  |  |
| No | 1 |  |
| Yes | 2.67 (1.79~3.96) | <0.001 |
| Coronary heart disease, n(%) |  |  |
| No | 1 |  |
| Yes | 2.55 (1.71~3.82) | <0.001 |
| Angina, n (%) |  |  |
| No | 1 |  |
| Yes | 2.6 (1.74~3.9) | <0.001 |
| Diabetes, n (%) |  |  |
| No | 1 |  |
| Yes | 2.02 (1.64~2.5) | <0.001 |
| Diabetes family history, n (%) |  |  |
| No | 1 |  |
| Yes | 0.8 (0.67~0.96) | 0.018 |
| Hyperlipidemia, n(%) |  |  |
| No | 1 |  |
| Yes | 2.1 (1.75~2.52) | <0.001 |
| Hormone replacement therapy, n (%) |  |  |
| No | 1 |  |
| Yes | 1.37 (1.04~1.8) | 0.024 |
| MCV, Mean ± SD | 1.05 (1.03~1.06) | <0.001 |
| Total cholesterol(mg/dL) | 1 (1~1.01) | 0.001 |
| High density lipoprotein | 1.01 (1~1.01) | 0.009 |
| HbA1c (%) | 1.17 (1.1~1.24) | <0.001 |
| Hemoglobin (mg/dL) | 1.02 (0.94~1.09) | 0.656 |
| Albumin, (mg/dL) | 0.64 (0.48~0.86) | 0.003 |
| Serum creatinine (mg/dL) | 1.31 (1.16~1.47) | <0.001 |
| eGFR, Mean ± SD | 0.97 (0.97~0.98) | <0.001 |
| rdw | 1.07 (1.01~1.12) | 0.017 |

**Table 2. Breast Cancer Covariate Screening and Collinearity Analysis.**

| Term1 | coeff1 | Change.  percentage1 | Term2 | coeff2 | Change.  percentage2 | GVIF | DF | colinearity | select |
| --- | --- | --- | --- | --- | --- | --- | --- | --- | --- |
| Crude model | 0.06 | Ref. | Full model | 0.06 | Ref. | Aliased variables exist | NA | NA | Ref. |
| Age | 0.03 | -45.3 | Age | 0.1 | 66.8 | Aliased variables exist | NA | NA | Yes |
| Race | 0.1 | 58.2 | Race | 0.06 | 2.3 | Aliased variables exist | NA | NA | Yes |
| Education levels | 0.07 | 3.3 | Education levels | 0.06 | 3.9 | Aliased variables exist | NA | NA | No |
| Marital, | 0.06 | -12.9 | Marital, | 0.06 | -2.7 | Aliased variables exist | NA | NA | Yes |
| PIR | 0.08 | 20.5 | PIR | 0.05 | -9.8 | Aliased variables exist | NA | NA | Yes |
| BMI | 0.07 | 12.6 | BMI | 0.06 | -0.9 | Aliased variables exist | NA | NA | Yes |
| Hypertensive | 0.04 | -38.5 | Hypertensive | 0.06 | 0.7 | Aliased variables exist | NA | NA | Yes |
| Heart failure | 0.05 | -17.5 | Heart failure | 0.06 | 1 | Aliased variables exist | NA | NA | Yes |
| Coronary heart disease | 0.06 | -6.9 | Coronary heart disease | 0.06 | -1.1 | Aliased variables exist | NA | NA | No |
| Angina | 0.06 | -3.6 | Angina | 0.06 | -1.4 | Aliased variables exist | NA | NA | No |
| Diabetes | 0.05 | -27.4 | Diabetes | 0.06 | 0.9 | Aliased variables exist | NA | NA | Yes |
| Diabetes family history | 0.07 | 4.6 | Diabetes family history | 0.06 | -0.7 | Aliased variables exist | NA | NA | No |
| Hyperlipidemia | 0.07 | 5.8 | Hyperlipidemia | 0.06 | -0.2 | Aliased variables exist | NA | NA | No |
| Hormone replacement therapy | 0.07 | 11 | Hormone replacement therapy | 0.07 | 14.3 | Aliased variables exist | NA | NA | Yes |
| MCV | 0.17 | 162.4 | MCV | 0.05 | -20.7 | Aliased variables exist | NA | NA | Yes |
| Total cholesterol | 0.07 | 11.1 | Total cholesterol | 0.06 | 1.9 | Aliased variables exist | NA | NA | Yes |
| High density lipoprotein | 0.07 | 5 | High density lipoprotein | 0.06 | -3.4 | Aliased variables exist | NA | NA | No |
| HbA1c | 0.05 | -15.2 | HbA1c | 0.06 | 0.1 | Aliased variables exist | NA | NA | Yes |
| Hemoglobin | 0.09 | 40.7 | Hemoglobin | 0.06 | 3.6 | Aliased variables exist | NA | NA | Yes |
| Albumin | 0.05 | -24.4 | Albumin | 0.06 | 6.8 | Aliased variables exist | NA | NA | Yes |
| Serum creatinine | 0.05 | -19 | Serum creatinine | 0.06 | -2.3 | Aliased variables exist | NA | NA | Yes |
| eGFR | 0.01 | -91.8 | eGFR | 0.06 | 1.1 | Aliased variables exist | NA | NA | Yes |

**Comment 2.2: In particular, variables such as HbA1c, HDL-C, and serum creatinine may function as mediators of the RDW–mortality relationship rather than confounders.**

**Response 2.2：**

Thank you for your insightful comment. In response to your query, we have conducted separate mediation analyses to examine the potential mediating roles of HbA1c, HDL-C, and serum creatinine in the relationship between RDW (red cell distribution width) and all-cause mortality. The results of these analyses are presented in **Table 3, Table 4, and Table 5,** respectively.

Our findings indicate that these variables do not serve as mediators in the RDW-mortality relationship. The mediation effects were found to be non-significant, suggesting that the associations observed are not mediated through these biochemical markers. They may be more likely to act as confounders or to have indirect associations with the outcome.

**Table 3. Mediation Analysis Results of HbA1c in the Relationship between RDW and All-Cause Mortality**

| Item | Mediation Effect |
| --- | --- |
| Average Causal Mediation Effects | 0 |
| Average Direct Effects | 0.0054 |
| Total Effect | 0.0053 |

Model 3: adjusted for Age, Sex, Race, Martial status, PIR, BMI, Hypertensive, Cardiovascular disease, angina, heart failure, diabetes, family history of diabetes, Hyperlipidemia, Total cholesterol, Albumin, Hemoglobin, MCV, eGFR, HDL, Serum Creatinine.

**Table 4. Mediation Analysis Results of HDL-C in the Relationship between RDW and All-Cause Mortality**

| Item | Mediation Effect |
| --- | --- |
| Average Causal Mediation Effects | 0 |
| Average Direct Effects | 0.0053 |
| Total Effect | 0.0053 |

Model 3: adjusted for Age, Sex, Race, Martial status, PIR, BMI, Hypertensive, Cardiovascular disease, angina, heart failure, diabetes, family history of diabetes, Hyperlipidemia, Total cholesterol, Albumin, Hemoglobin, MCV, HbA1c, eGFR, Serum Creatinine.

**Table 5. Mediation Analysis Results of Serum Creatinine in the Relationship between RDW and All-Cause Mortality**

| Item | Mediation Effect |
| --- | --- |
| Average Causal Mediation Effects | 0.0005 |
| Average Direct Effects | 0.001 |
| Total Effect | 0.0015 |

Model 3: adjusted for Age, Sex, Race, Martial status, PIR, BMI, Hypertensive, Cardiovascular disease, angina, heart failure, diabetes, family history of diabetes, Hyperlipidemia, Total cholesterol, Albumin, Hemoglobin, MCV, HbA1c, HDL-C, eGFR.

**Comment 2.3：clarify whether model assumptions (such as the proportional hazards assumption) were assessed and met.**

**Response 2.3：**

Thank you for your valuable feedback. In response to your question regarding the assessment of model assumptions, including the proportional hazards assumption, we have conducted a thorough analysis.

We have performed tests to evaluate the proportional hazards assumption for our models, focusing on two types of mortality outcomes: all-cause mortality and cardiovascular disease (CVD) mortality. The results of these tests are summarized in the two tables provided.

For both RDW and GLOBAL variables across both mortality types, the p-values obtained from the proportional hazards tests are well above the conventional threshold of 0.05. Specifically, for all-cause mortality, the p-values are 0.795 for both RDW and GLOBAL **(Table 6)**. For CVD mortality, the p-values are 0.934 for both RDW and GLOBAL **(Table 7)**. These results indicate that we fail to reject the null hypothesis that the proportional hazards assumption holds.

Therefore, based on these findings, we conclude that the proportional hazards assumption is met for the models analyzed. This supports the validity of our model estimates and the interpretations drawn from them.

We appreciate your attention to this critical aspect of our analysis and hope that the additional information provided satisfies your query.

**Table 6. Assessment of Proportional Hazards Assumption for All-Cause Mortality**

| Variable | Chi-squared statistic | Degrees of Freedom | p.value |
| --- | --- | --- | --- |
| RDW | 0.067 | 1 | 0.795 |
| GLOBAL | 0.067 | 1 | 0.795 |

**Table 7. Assessment of Proportional Hazards Assumption for Cardiovascular Disease (CVD) Mortality**

| Variable | Chi-squared statistic | Degrees of Freedom | p.value |
| --- | --- | --- | --- |
| RDW | 0.007 | 1 | 0.934 |
| GLOBAL | 0.007 | 1 | 0.934 |

**Comment 3. Handling of Missing Data The decision to exclude individuals with missing covariate values may introduce bias and reduce generalizability. Please report the extent of missing data for key variables and justify your approach. If feasible, consider applying multiple imputation or conducting a sensitivity analysis to assess whether your findings are robust to missing data assumptions. Also the use of several statistical methods (Cox regression, Kaplan-Meier, RCS, time-dependent ROC) is appreciated but increases the risk of type I error. Please clarify whether any adjustments for multiple comparisons were applied. In addition, we encourage inclusion of model diagnostics, internal validation methods, or sensitivity analyses to evaluate the robustness of your findings.**

**Comment 3.1: The decision to exclude individuals with missing covariate values may introduce bias and reduce generalizability. Please report the extent of missing data for key variables and justify your approach.**

**Response 3.1：**

We sincerely appreciate the reviewer's insightful comment regarding missing data of covariates.

In response to the concern regarding the exclusion of individuals with missing covariate values, we have carefully assessed the extent of missing data in our dataset. After excluding cases with missing values, the sample size was reduced to 15,806. Our analysis revealed that the poverty-income ratio (PIR) variable had the highest missing rate (9.042%), while all other covariates demonstrated minimal missingness (<5%) **(Table 8)**. Given that the overall missingness is relatively low (<10%for the most affected variable), we initially employed a complete-case analysis by excluding individuals with missing covariate values. This approach is commonly used in the literature when missing data are minimal, as it helps to maintain the simplicity and interpretability of the analysis. Numerous relevant studies on RDW have employed the complete-case analysis method, directly excluding cases with missing covariate values for statistical analysis[1,6].

To ensure robust results and minimize potential bias, we will implement multiple imputation techniques for missing data handling, followed by comprehensive sensitivity analyses to validate the stability of our findings. In contrast, following the application of multiple imputation, the sample size increased to 22,726. **(Table 9)**

**Table 8. Missing data statistics of key variables**

| Covariate | Missing Frequency | Missing Percentage |
| --- | --- | --- |
| Age | 0 | 0 |
| Race | 0 | 0 |
| Education | 34 | 0.1496 |
| Marital | 216 | 0.9505 |
| PIR | 2055 | 9.0425 |
| BMI | 365 | 1.6061 |
| Hypertension | 0 | 0 |
| Heart failure | 53 | 0.2332 |
| Cardiovascular disease | 82 | 0.3608 |
| Angina | 65 | 0.286 |
| Diabetes | 0 | 0 |
| Diabetes family history | 399 | 1.7557 |
| Hyperlipidemia | 0 | 0 |
| Hormone replacement  therapy | 0 | 0 |
| Mcv | 0 | 0 |
| Total cholesterol | 400 | 1.7601 |
| High density lipoprotein | 401 | 1.7645 |
| HbA1c | 70 | 0.308 |
| Hemoglobin | 0 | 0 |
| Albumin | 467 | 2.0549 |
| Serum creatinine | 469 | 2.0637 |
| eGFR | 469 | 2.0637 |

**Comment 3.2: Also the use of several statistical methods (Cox regression, Kaplan-Meier, RCS, time-dependent ROC) is appreciated but increases the risk of type I error. Please clarify whether any adjustments for multiple comparisons were applied. In addition, we encourage inclusion of model diagnostics, internal validation methods, or sensitivity analyses to evaluate the robustness of your findings.**

**Response 3.2:**

In this study, multiple statistical methods, including Cox regression, Kaplan–Meier curves, restricted cubic splines (RCS), and time-dependent receiver operating characteristic (ROC) analyses, were employed to investigate the relationship between red blood cell distribution width (RDW) and all-cause as well as cardiovascular mortality in patients with breast cancer. In the original analyses, cases with missing values were directly excluded to ensure data completeness for the initial assessment (see **original** **Table 1, Table 2, Figure 1, Figure 2, and Figure 4**). To assess the robustness of the findings in the presence of missing data, multiple imputation was employed to fill in the missing values. The analyses were then repeated using the imputed datasets to evaluate the consistency and stability of the results (see **Table 9, Table 10, Figure 2, Figure 3, and Figure 4**).

**Baseline characteristics**: The analysis revealed that the proportions of breast cancer cases (3.0% in both scenarios), all-cause mortality (13.2% vs. 13.1%), and cardiovascular mortality (4.2% in both) were remarkably consistent between the datasets with missing values excluded and those with multiple imputation applied. In contrast, the mean follow-up time showed a notable difference, being 126.9 months in the excluded dataset versus 115.5 months in the imputed dataset (Table 1 original data VS Table 9). The distributions of breast cancer incidence, all-cause mortality, and cardiovascular mortality were largely consistent between the analyses with missing values excluded and those with multiple imputation applied, underscoring the robustness of the findings.

**Cox regression**: In the Cox regression analysis of all-cause mortality without multiple imputation **(Table 2 original data)**, the hazard ratio (HR) for RDW in the crude model was 1.39 (95% CI: 1.25–1.54), and after full adjustment in Model 3, it increased to 1.56 (95% CI: 1.35–1.80). For the RDW group, the crude model showed an HR of 2.39 (95% CI: 1.66–3.45) for the high RDW group (≥12.68%) compared to the low RDW group (<12.68%), which attenuated to 2.13 (95% CI: 1.42–3.20) in Model 3.After multiple imputation **(Table 10)**, the HR for RDW in the crude model was lower at 1.24 (95% CI: 1.16–1.34) and slightly increased to 1.25 (95% CI: 1.12–1.38) in Model 3. For the RDW group, the crude model HR for the high RDW group was 1.82 (95% CI: 1.36–2.45), attenuating to 1.46 (95% CI: 1.06–2.02) in Model 3. The trend for cardiovascular mortality after multiple imputation is consistent with that before multiple imputation. Although the specific values have changed, the risk of mortality in the high RDW group remains significantly higher than that in the low RDW group.

**RCS:** The restricted cubic spline (RCS) analysis visualizing the relationship between RDW and all-cause mortality (Panel A) as well as cardiovascular mortality (Panel B) among breast cancer patients demonstrated a linear association in both the original dataset with missing values excluded and the dataset after multiple imputation (p for non-linearity > 0.05), indicating a consistent trend. **(Figure 1 original data VS Figure 2)**

**Kaplan-Meier:** we employed Kaplan–Meier survival curves to elucidate the relationship between RDW and both all-cause and cardiovascular mortality in our breast cancer cohort. Notably, the Kaplan–Meier curves derived from the dataset with missing values excluded **(Figure 2 original data)** and those from the dataset following multiple imputation **(Figure 3)** consistently demonstrated that patients in the low RDW group (<12.68%) exhibited significantly higher survival rates compared to those in the high RDW group (≥12.68%). This consistent trend across both analytical approaches underscores RDW's robustness as a prognostic indicator of mortality risk.

**Subgroup Analyses:** In the subgroup analyses for all-cause mortality stratified by age, BMI, diabetes, and eGFR, no significant interaction effects were observed (p > 0.05) following the handling of missing covariate values and multiple imputation. This indicates consistent results across all stratified analyses. **(Figure 3 original data VS Figure 4).**

**time-dependent ROC:** The stability in AUC values for all-cause mortality both before and after multiple imputation suggests that RDW is a robust predictor for this outcome. The predictive ability of RDW for all-cause mortality remains consistent over time, regardless of the data handling method. This consistency underscores the reliability of RDW as a prognostic marker for all-cause mortality. **(Figure 4 A, B original data VS Figure 5 A, B).**

The observed trend of a slight decline in AUC values for cardiovascular mortality (CVD-mortality) when excluding missing values, versus an increasing trend in AUC values after multiple imputation, may be attributed to the larger sample size achieved through imputation. As more events accumulate over time in the larger dataset, the model's capacity to distinguish between higher and lower risks is enhanced, thereby leading to the upward trend in AUC values. **(Figure 4 C,D original data VS Figure 5 C,D).**

**Assessment of Model Performance: ROC, AUC, and C-index Comparisons between Model 1 and Model 1+RDW**

**After Multiple Imputation (Figure 6):**

The inclusion of RDW in Model 1+RDW slightly enhances discriminative ability for both all-cause and CVD mortality, as shown by ROC curves closer to the top-left corner. (**Figure 6A**: All-cause mortality, **Figure 6B**: CVD mortality)

Model 1+RDW consistently outperforms Model 1 in AUC, indicating better discriminative performance for predicting all-cause and CVD mortality. (**Figure 6C**: All-cause mortality, **Figure 6D**: CVD mortality)

C-index Values (Panels E & F): The C-index for Model 1+RDW is higher than Model 1, suggesting improved risk ranking for all-cause and CVD mortality over time. (**Figure 6E**: All-cause mortality, **Figure 6F**:CVD mortality)

Adding RDW to the model (Model 1+RDW) results in a modest but significant improvement in predictive performance for both all-cause and CVD mortality, as evidenced by higher AUC and C-index values.

**After excluding individuals with missing covariate (Figure 7):**

Model 1+RDW demonstrated a slight improvement in discriminative ability for both all-cause mortality **(Figure 7A)** and CVD mortality **(Figure 7B)**, as indicated by the ROC curves. The AUC values **(Figure 7C, 7D)** consistently favored Model 1+RDW over Model 1, suggesting enhanced predictive performance for both mortality outcomes. The C-index further confirmed the modest but significant advantage of incorporating RDW into the model for predicting mortality risks over time. **(Figure 7E, 7F)**

In summary, the inclusion of RDW in predictive models consistently enhances their performance in forecasting all-cause and cardiovascular disease mortality, regardless of the method used to handle missing covariate data.

**Table 9 Baseline characteristics of critical patients with low and high RDW after multiple imputation**

| **Characteristic** | **Total** | **Low RDW** | **High RDW** | **p** |
| --- | --- | --- | --- | --- |
|  |  | **(<12.68%)** | **(≥12.68%)** |  |
| Samplesize,N | 22726 | 8489 | 14237 |  |
| Age(years) | 49.7±17.8 | 46.5±17.4 | 51.6±17.8 | <0.001 |
| Race,n(%) |  |  |  | <0.001 |
| Non-Hispanic White | 4078(17.9) | 1666(19.6) | 2412(16.9) |  |
| Non-Hispanic Black | 2094(9.2) | 676(8) | 1418(10) |  |
| Mexican American | 9500(41.8) | 4334(51.1) | 5166(36.3) |  |
| Other | 7054(31.0) | 1813(21.4) | 5241(36.8) |  |
| Education,n(%) |  |  |  | <0.001 |
| blow high school | 5996(26.4) | 2035(24) | 3961(27.9) |  |
| high school | 5095(22.5) | 1874(22.1) | 3221(22.7) |  |
| above high school | 11601(51.1) | 4573(53.9) | 7028(49.5) |  |
| Marital,n(%) |  |  |  | <0.001 |
| Married | 12205(54.2) | 4917(58.8) | 7288(51.5) |  |
| Living alone | 6468(28.7) | 1973(23.6) | 4495(31.8) |  |
| Never married | 3837(17.0) | 1471(17.6) | 2366(16.7) |  |
| PIR,n(%) |  |  |  | <0.001 |
| Low | 6810(32.9) | 2190(28.1) | 4620(35.9) |  |
| Medium | 7807(37.8) | 2877(36.9) | 4930(38.3) |  |
| High | 6054(29.3) | 2721(34.9) | 3333(25.9) |  |
| BMI(kg/m2) | 29.4±7.5 | 27.5±6.1 | 30.5±8.0 | <0.001 |
| Hypertensive,n(%) |  |  |  | <0.001 |
| No | 13209(58.1) | 5651(66.6) | 7558(53.1) |  |
| Yes | 9517(41.9) | 2838(33.4) | 6679(46.9) |  |
| Heart failure,n(%) |  |  |  | <0.001 |
| No | 22076(97.4) | 8382(98.9) | 13694(96.5) |  |
| Yes | 597(2.6) | 93(1.1) | 504(3.5) |  |
| Coronary heart disease,n(%) |  |  |  | <0.001 |
| No | 22075(97.5) | 8335(98.4) | 13740(96.9) |  |
| Yes | 569(2.5) | 133(1.6) | 436(3.1) |  |
| Angina,n(%) |  |  |  | <0.001 |
| No | 22111(97.6) | 8317(98.2) | 13794(97.2) |  |
| Yes | 550(2.4) | 152(1.8) | 398(2.8) |  |
| Diabetes,n(%) |  |  |  | <0.001 |
| No | 19131(84.2) | 7562(89.1) | 11569(81.3) |  |
| Yes | 3595(15.8) | 927(10.9) | 2668(18.7) |  |
| Diabetes family history,n(%) |  |  |  | <0.001 |
| No | 11769(52.7) | 4598(55.2) | 7171(51.3) |  |
| Yes | 10558(47.3) | 3738(44.8) | 6820(48.7) |  |
| Hyperlipidemia,n(%) |  |  |  | <0.001 |
| No | 14055(61.8) | 5535(65.2) | 8520(59.8) |  |
| Yes | 8671(38.2) | 2954(34.8) | 5717(40.2) |  |
| Hormonere placement therapy,n(%) |  |  |  | <0.001 |
| No | 20872(91.8) | 7445(87.7) | 13427(94.3) |  |
| Yes | 1854(8.2) | 1044(12.3) | 810(5.7) |  |
| MCV,Mean±SD | 88.7±6.2 | 91.0±4.1 | 87.3±6.8 | <0.001 |
| Total cholesterol(mg/dL) | 197.4±41.0 | 198.8±40.1 | 196.5±41.5 | <0.001 |
| High density lipoprotein(mg/dL) | 57.2±16.2 | 57.6±15.6 | 57.1±16.5 | 0.025 |
| HbA1c(%) | 5.7±1.1 | 5.5±1.0 | 5.8±1.1 | <0.001 |
| Hemoglobin(mg/dL) | 13.3±1.3 | 13.7±1.0 | 13.1±1.3 | <0.001 |
| Albumin,(mg/dL) | 4.2±0.3 | 4.2±0.3 | 4.1±0.3 | <0.001 |
| Serum creatinine(mg/dL) | 0.8±0.4 | 0.7±0.2 | 0.8±0.5 | <0.001 |
| eGFR,Mean±SD | 95.8±23.3 | 100.0±21.0 | 93.2±24.3 | <0.001 |
| Breast cancer ,n(%) |  |  |  | <0.001 |
| No | 22040 (97.0) | 8306 (97.8) | 13734 (96.5) |  |
| Yes | 686 ( 3.0) | 183 (2.2) | 503 (3.5) |  |
| All-cause mortality,n(%) |  |  |  | <0.001 |
| No | 19757(86.9) | 7537(88.8) | 12220(85.8) |  |
| Yes | 2969(13.1) | 952(11.2) | 2017(14.2) |  |
| CVD mortality,n(%) |  |  |  | <0.001 |
| No | 21769(95.8) | 8202(96.6) | 13567(95.3) |  |
| Yes | 957(4.2) | 287(3.4) | 670(4.7) |  |
| Follow-uptime(months) | 115.5±65.9 | 147.3±58.5 | 96.5±62.6 | <0.001 |

**Table 10. Association between RDW and RDW group and breast cancer in multiple regression model after multiple imputation**

| **Variable** | **HR (95%CI), P value** | | | |
| --- | --- | --- | --- | --- |
|  | **Crude** | **Model 1** | **Model 2** | **Model 3** |
| **All-cause mortality** |  |  |  |  |
| RDW | 1.24 (1.16~1.34) <0.001 | 1.19 (1.09~1.29) <0.001 | 1.18 (1.08~1.28) <0.001 | 1.25 (1.12~1.38) <0.001 |
| RDW group |  |  |  |  |
| Low RDW | Reference | Reference | Reference | Reference |
| (<12.68%) |  |  |  |  |
| High RDW | 1.82 (1.36~2.45) <0.001 | 1.49(1.1~2.02)0.009 | 1.47(1.08~2.01) 0.014 | 1.46(1.06~2.02) 0.02 |
| (≥12.68%) |  |  |  |  |
| **Cardiovascular mortality** |  |  |  |  |
| RDW | 1.32(1.16~1.5) <0.001 | 1.3(1.11~1.53) 0.001 | 1.3(1.1~1.54) 0.002 | 1.35(1.07~1.7) 0.01 |
| RDW group |  |  |  |  |
| Low RDW | Reference | Reference | Reference | Reference |
| (<12.68%) |  |  |  |  |
| High RDW | 2.71(1.43~5.15) 0.002 | 2.18(1.14~4.18) 0.019 | 2.37(1.2~4.68) 0.013 | 2.12(1.05~4.29) 0.037 |
| (≥12.68%) |  |  |  |  |

**Figure 2. A linear relationship between RDW with all-cause (A) and cardiovascular mortality (B) among breast cancer visualized by restricted cubic spline （after multiple imputation）**

**Figure 3. Kaplan–Meier curves of the survival rate with higher (<12.68%) and lower (≥12.68%) RDW values.（after multiple imputation）**

**Figure 4. Forest plot depicting the association between the RDW and the risk of all-cause mortality （after multiple imputation）**

Abbreviation: PIR: Poverty income ratio; BMI: body mass index; eGFR: estimated glomerular filtration rate; RDW, red blood cell distribution width.

**Figure 5. Time-dependent ROC curves and time-dependent AUC values**

**Time-dependent ROC curves and time-dependent AUC values (with 95% confidence band) of the RDW for predicting all-cause mortality (A, B), cardiovascular mortality (C, D). （after multiple imputation）**

**Figure 6. Assessment of Model Performance: ROC, AUC, and C-index Comparisons between Model 1 and Model 1+RDW (after multiple imputation)**

**Figure 7. Assessment of Model Performance: ROC, AUC, and C-index Comparisons between Model 1 and Model 1+RDW (After excluding individuals with missing covariate)**

**2、Independent Review Report, Reviewer 1**

**Response to Reviewer 1:**

**Comment 1: On line 114 you say 'available at' but the sentence is incomplete.**

**Response 1:**

Thank you for pointing out the incomplete sentence. Mortality data for the study population were sourced through linkage between NHANES and the National Death Index (NDI), available at https://www.cdc.gov/nchs/data-linkage/mortality.htm.

*Revised Manuscript at Line113-114:*

*Mortality data for the study population were sourced through linkage between NHANES and the National Death Index (NDI), available at https://www.cdc.gov/nchs/data-linkage/mortality.htm.*

**Comment 2:The results are interesting, and should be useful to those working in the field. The English language is clear. The Figures are clear.**

**Response 2:**

Thank you very much for your positive feedback and for finding our results interesting and potentially useful to the field. We are delighted to hear that you find the English language clear and the figures easy to understand. Your comments are greatly appreciated and will help us in finalizing the manuscript. We look forward to making any necessary revisions and are grateful for your support and encouragement.

**3、Independent Review Report, Reviewer 2**

**Reviewer 2: General**

**Comment 1: This study aims to determine the association between RDW and all-cause or cardiovascular mortality in 476 breast cancer patients in a cohort study using the NHANES data compared to 15,806 patients without breast cancer.**

**Response 1:**

Thank you for your thorough review and for highlighting the importance of clarity in our study's description. You are correct, and we appreciate your attention to detail. We have made the necessary correction to accurately reflect the total number of participants in our study.

The revised statement is as follows:

"This study aims to determine the association between RDW and all-cause or cardiovascular mortality in a cohort of 476 breast cancer patients and 15,330 patients without breast cancer, making a total of 15,806 participants in the NHANES data." And Figure S1 has been revised.

We apologize for any confusion caused by the initial oversight and are grateful for the opportunity to clarify this point.

**Comment 2: RDW alone will hardly be used or useful in the setting of mortality. However, you may want to prominently discuss the strength of your approach: In these times of big data, RWD may be an inexpensive, simple and seemingly unspecific, yet surprisingly informative parameter during the comprehensive evaluation of patient data to predict outcome.**

**Response 2：**

We sincerely appreciate the thoughtful comments from the reviewer regarding the clinical utility of RDW. While we concur that RDW alone may not suffice as an independent mortality predictor, our study underscores its unique strengths as a cost-effective, universally available, and comprehensive biomarker within an integrated patient assessment framework.

RDW is an inexpensive, straightforward, and information-rich parameter, routinely measured as part of a standard complete blood count (CBC) without the need for additional tests or substantial costs. Its measurement does not rely on complex equipment or techniques and can be performed in any healthcare setting that conducts routine blood tests, ensuring high global accessibility.

Despite its seemingly nonspecific nature, RDW provides valuable insights into a patient's inflammatory status[12,13], nutritional condition[14], and potential cardiovascular risks[1,15,16]. Its association with various adverse health outcomes, including mortality, makes it a powerful predictive tool.

In the era of big data, the simplicity of RDW allows for rapid risk stratification prior to the deployment of expensive/invasive tests. RDW can be effectively integrated with other extensive datasets to enhance the accuracy and reliability of predictive models.

*Revised Manuscript at Line 351(Discussion):*

Furthermore, the consistency of results across various subgroups without significant interaction effects reinforces the reliability of RDW as a mortality predictor in diverse patient populations.

*In today's era of big data, the simplicity of RDW allows for rapid risk stratification prior to the deployment of expensive/invasive tests. RDW can be effectively integrated with other extensive datasets to enhance the accuracy and reliability of predictive models.*

**Comment 3: It is refreshing to see how researchers from China help to utilize and analyze data from a US-based survey of 20 years, hinting to the power of publically available datasets. More such crossborder transperancy could benefit all.**

**Response 3:**

Thank you for your encouraging comment and for recognizing the value of our cross-border research efforts. We are delighted to hear that you find our use of the US-based survey data from NHANES refreshing and powerful. The sharing and analysis of publicly available datasets can lead to new insights and a better understanding of global health issues.

**Comment 4: The English is good and the use of AI to 'polish' the manuscript appreciated (line 373). Please be specific what exact tool(s) have been used (line 374).**

**Response 4：**

Thank you for your careful reading and question. As requested, we clarify that the AI-driven polishing was performed using ChatGPT (OpenAI GPT-4 version) for grammar, syntax refinement, and fluency enhancements, with all edits manually reviewed to ensure accuracy.

**Comment 5: I would recommend to focus on the immediate observation and shorten the manuscript substantially, particularly its Discussion. This sharpening of the message could enhance the quality for the readers.**

**Response 5:**

We thank the reviewer for the constructive suggestion. As recommended, we have substantially shortened the Discussion section (now reduced by 10%), focusing on the key findings and their immediate implications. Redundant interpretations and tangential comparisons were removed to sharpen the manuscript’s message.

**Specific**

**Comment 6: I would not call this a "relationship" (line 23). Rather, it is a correlation of a surrogate marker (RDW) with highly clinically relevant outcome parameters. The "strong predictive power" (line 47) is, unfortunately, of little practical utility for now. What would you recommend to do once the RDW is determined? Also: how strong is "strong" and when, compared to which other paraameters, is RWD a stronger parameter?**

**Comment 6.1:** I would not call this a "relationship" (line 23). Rather, it is a correlation of a surrogate marker (RDW) with highly clinically relevant outcome parameters.

**Response 6.1**：

We appreciate the reviewer’s precise comment. As suggested, we have revised the phrasing to clarify that RDW serves as a surrogate marker correlated with clinical outcomes (rather than implying a direct mechanistic relationship).

The updated text now reads:

*The correlation between red cell distribution width (RDW) and mortality in breast cancer participants remains poorly characterized.*

**Comment 6.2:** The "strong predictive power" (line 47) is, unfortunately, of little practical utility for now. What would you recommend to do once the RDW is determined?

**Response 6.2:**

We appreciate the reviewer's question regarding the practical application of RDW. While RDW alone is not a diagnostic tool, its prognostic value.

①In our clinical practice, RDW, as a routinely available parameter in complete blood counts (CBC), serves as a cost-effective and accessible prognostic biomarker for breast cancer patients. We recommend incorporating RDW into existing risk stratification frameworks (such as: Nottingham Prognostic Index) to enhance risk assessment, particularly in resource-limited settings where advanced biomarkers are unavailable. ②In cases of elevated RDW, such as values greater than or equal to 12.68%, clinicians should conduct a comprehensive evaluation for underlying conditions associated with poor outcomes, including anemia, chronic inflammation, and malnutrition. Additionally, they should implement intensified monitoring protocols, including shortened follow-up intervals and proactive management of comorbidities, to mitigate mortality risks. ③Given the non-specificity of RDW, future research should focus on developing composite prognostic scores that combine RDW with imaging or omics-based biomarkers to improve specificity.

**Comment 6.3:** Also: how strong is "strong" and when, compared to which other parameters, is RWD a stronger parameter?

**Response 6.3:**

We sincerely appreciate the reviewer's insightful questions regarding the clinical utility and predictive strength of RDW.

The term "strong" in describing RDW's predictive value is supported by evidence from our analyses:1) For short-term prediction (1-3 years), RDW demonstrated excellent discriminative ability with AUC values of 0.85-0.71 for all-cause mortality and 0.91-0.82 for cardiovascular mortality **(Figure 4 A, C original data)**. 2) For long-term prognosis spanning 5 to 10 years, RDW exhibits moderate yet clinically significant predictive power, with AUC values ranging from 0.64 to 0.69 for all-cause mortality and from 0.57 to 0.70 for cardiovascular mortality. While the graphs (A and B for all-cause mortality, C and D for cardiovascular mortality) do not directly compare RDW with other parameters, the high AUC values suggest that RDW is a strong independent predictor.

**Comment 7: RDW groups (line 189) are at the core of the study and need to be better defined. There may be dynamic changes of RDW at multiple time points. Define when exactly RDW was measured. What RDW did you use if there are several RDW tests with differing results?**

**Response 7:**

We thank the reviewer for this important methodological question. In our study, RDW values were obtained during the NHANES examination visit when participants self-reported their breast cancer diagnosis through the Medical Conditions Questionnaire (MCQ). Since NHANES is a cross-sectional survey, only baseline RDW measurements were available, and we used the single RDW value collected at this initial examination for all analyses.

As the editor pointed out in their first comment, I have provided clarifications in the "Materials and Methods—Ascertainment of RDW" section. The revised document is as follows.

*Revised Manuscript at Lines 121-125:*

**Ascertainment of RDW**

*In our study, RDW was measured only at baseline during the initial examination visit, and no repeated measurements were conducted over time. Red cell distribution width (RDW) determination was performed based on the full blood count data, utilizing a Beckman Coulter MAXM automated hematology analyzer to measure the values.*

**Comment 8: You document carefully the many parameters (lines 201 to 203, Table 1 and Table S1) that are significantly correlated with RWD. These other parameters, such as age, race, education and so on, may well influence the outcome. What exactly does RWD add? Where do you prove the added benefit of testing RWD and how are you taking RWD in account?**

**Response 8：**

We are grateful for the reviewer's insightful comments and questions, which have provided an opportunity to further clarify the role and significance of Red Cell Distribution Width (RDW) in our study.

In **Table 1**, patients were categorized into low and high RDW groups, allowing us to examine the distribution of various independent variables such as age, race, and education. This categorization revealed significant differences in these variables between the RDW groups (p < 0.001 for most variables). **Table S1**, on the other hand, presents the distribution of these variables based on the presence or absence of breast cancer. This comparison highlights differences in baseline characteristics between patients with and without breast cancer. The statistical significance of RDW is clearly demonstrated by the p-values presented in both **Table 1 and Table S1**. The RDW groups show significant differences in outcomes, such as all-cause mortality and cardiovascular mortality. Moreover, the consistent association of RDW with adverse outcomes across different patient subgroups, including those with and without breast cancer, underscores its clinical relevance. This initial descriptive analysis lays the groundwork for subsequent statistical modeling.

Our analysis progressed from a crude model to increasingly adjusted models (Model 1 to Model 3), each incorporating more covariates to control for potential confounding factors. This stepwise approach allowed us to isolate the effect of RDW on outcomes while accounting for other influential variables. The consistent significance of RDW across these models further validates its role as an independent predictor.

**Comment 9: Line 282: "a likely independent forecaster" - what is the proof of being independence (and independent of what exact other parameters)?**

**Response 9:**

We sincerely appreciate the opportunity to clarify this important methodological consideration. Upon careful re-evaluation, we recognize that our original phrasing ("likely independent") did not fully capture the robust statistical evidence from the referenced study. The cited study [29] definitively established RDW as an prognostic factor for short-to-medium term mortality (6-36 months) in critically ill breast cancer patients from the MIMIC-IV cohort, demonstrating strong associations after rigorous adjustment for ICU-specific confounders (6-month mortality: adjusted HR 3.20, 95% CI 1.75-5.76, p < 0.001).

We have revised our text to more accurately reflect these findings: "In a critically ill breast cancer cohort, elevated RDW showed a strong independent association with short-term mortality (adjusted HR 3.20 for 6-month mortality) [29]."

**Original version Line 282:**

“In a cohort of 939 women with breast cancer, high RDW was found to be a likely independent forecaster of greater all-cause mortality within the first 6 months, 1 year, and 3 years following identification [29]”

***Revised Text for Line 282:***

*In a cohort of 939 women with breast cancer,* *elevated RDW demonstrated strong independent prognostic value for all-cause mortality within the first 6 months, 1 year, and 3 years following identification[29].*

**Comment 10: RDW is a surrogate marker and iany possible mechanistic link to outcome is lacking. It does not help when you explain the lack of a link by RWD's association with other surrogate markers (lines 292 - 296). Shorten these pseculations much.**

**Response：**

We sincerely appreciate the reviewer's valuable feedback. In response to your comments, we have completely removed the speculative content regarding potential mechanistic associations with other surrogate markers in Lines 292-296.

**Comment 11: Your "inability to establish a definitive causal link" (line 352) is not "due to its retrospective cohort design" (line 354). There may well be no definitive causal link at all. You do not make a good case that "prospective studies are necessary" (line 363 - 364) or helpful in any way.**

**Response:**

We sincerely appreciate the reviewer's critical feedback on our manuscript, particularly regarding the establishment of causality and the necessity of prospective studies.

We concur with the reviewer that the inability to establish a definitive causal link should not be simplistically attributed to the retrospective nature of our study. Retrospective studies can provide valuable insights into causal relationships, albeit with certain limitations. The true complexity lies in the potential for unmeasured confounding variables and the inherent challenges in temporality proof, which are not solely resolved by the study design. Our intent was not to suggest that retrospective studies are incapable of establishing causality but rather to acknowledge that our study's design presents certain constraints in proving causality definitively. To address these issues, we have revised our discussion accordingly.

Line 352-354:

The limitations of this study include the inability to establish a definitive causal link between elevated RDW and mortality in breast cancer patients due to its retrospective cohort design.

*The revisions are as follows:*

*Despite demonstrating a significant association between RDW and mortality, our observational study's findings are limited by potential residual confounding and the inability to establish causality due to unmeasured treatment details and temporal changes.*

Regarding the suggestion for prospective studies, we recognize that we did not adequately justify this recommendation in our original manuscript. We appreciate the reviewer's insight that prospective studies are not inherently more capable of establishing causality than retrospective studies. Our rationale for suggesting prospective studies was to highlight the potential benefits of observing temporal relationships more directly and to control for confounding factors more effectively through study design. However, we understand that this does not guarantee the establishment of causality.

We have removed the unqualified recommendation for prospective studies. The revised conclusion now focuses solely on our demonstrated findings regarding RDW's prognostic value in this cohort.

*The revisions are as follows:*

*RDW is an important independent indicator of both all-cause and cardiovascular mortality in breast cancer patients, emphasizing its potential as a simple and cost-effective biomarker for clinical risk assessment.*

**Comment 12: RDW is a measure of red cell volume heterogeneity, as such, red cell transfusion can be a confounding factor. How are you taking account of this possible effect? Compare PMID 29770452 and discuss.**

**Response:**

We appreciate the reviewer's insightful comments regarding the potential confounding effect of red blood cell (RBC) transfusions on Red Cell Distribution Width (RDW) values.

We acknowledge that RBC transfusions can significantly alter RDW values, as demonstrated in the study, which showed that RDW values increased after transfusion, with the highest level observed 24 hours post-transfusion[17].In our study, we did not have access to detailed data on RBC transfusions from the NHANES database, which indeed limits our ability to control for this potential confounding factor. We suggest that future studies incorporate detailed data on red blood cell transfusions and consider the timing of RDW measurements relative to transfusion events to more accurately assess the prognostic role of RDW. We will enhance the discussion on the limitations of our study, emphasizing the potential impact of red blood cell (RBC) transfusions and the need for caution when interpreting our results.

Line 352-359:

The limitations of this study include the inability to establish a definitive causal link between elevated RDW and mortality in breast cancer patients due to its retrospective cohort design. Secondly, despite adjusting for various confounding factors, residual confounding effects may persist, including those related to cancer treatment modalities and comorbidities that have not been fully addressed. Thirdly, measuring RDW at a single time point may not capture temporal fluctuations that could influence mortality risk. Furthermore, the study population was derived from NHANES data, which may restrict the generalizability of the findings to other populations with disparate characteristics.

*The revisions are as follows:*

*Despite demonstrating a significant association between RDW and mortality, our observational study's findings are limited by potential residual confounding and the inability to establish causality due to unmeasured treatment details and temporal changes.*

Second, although we adjusted for multiple confounders, residual confounding may persist due to unmeasured variables including those cancer treatment modalities, specific comorbidity severities, and RBC transfusion history - particularly relevant given established transfusion-induced RDW fluctuations*[17]*, which could not be addressed due to NHANES data limitations. Furthermore, the study population was derived from NHANES data, which may restrict the generalizability of the findings to other populations with disparate characteristics.

**Minor**

**There are many spots were refining and shortening of the text can contribute to focussing the manuscript on the main message.**

**Comment 13. It's not an "RWD level" (line 69), better: RWD parameter or, simply, "RWD".**

**Response 13：**

We sincerely appreciate the reviewer's meticulous attention to terminology accuracy.

Line 69: Increased RDW levels are linked to a range of conditions, including heart-related diseases (such as heart failure and

*As recommended, we have revised the text at line 69:*

*Increased RDW are linked to a range of conditions, including heart-related diseases (such as heart failure and coronary artery disease).*

**Comment 14. Add new paragraphs at lines 167 and 181.**

**Response 14:**

We sincerely appreciate the reviewer’s thoughtful suggestions to improve the organization and clarity of our statistical methods section. The request to add new paragraphs at lines 167 and 181 has helped enhance the logical flow and readability of our manuscript. As recommended, we have restructured the text by inserting two new paragraphs at the specified locations:

The χ² test was used to analyze categorical variables. Additionally, Cox proportional hazards regression models were applied to evaluate the correlation between red cell distribution width (RDW) and the incidence of breast cancer, taking into account various clinical features. Three distinct analytical models were constructed to account for demographic, socioeconomic, and clinical confounders.

*[New paragraph at line 167]*

Model 1 incorporated adjustments for basic demographic factors, including age, gender, ethnicity, marital status, and the poverty income ratio (PIR). Model 2 expanded on these adjustments by further accounting for BMI, hypertension, coronary heart disease, angina, heart failure, diabetes, family history of diabetes, hormone replacement therapy, and hyperlipidemia. Model 3 encompassed all variables in Model 2 and further refined the analysis by incorporating additional biochemical and hematological parameters, such as glycated hemoglobin (HbA1c), total cholesterol, high-density lipoprotein cholesterol (HDL-C), serum albumin, hemoglobin concentration, mean corpuscular volume (MCV), serum creatinine levels, and estimated glomerular filtration rate (eGFR).

*[New paragraph at line 181]*

To assess the nonlinear relationship between RDW and mortality risk, restricted cubic spline (RCS) analysis with three knots (10th, 50th, and 90th percentiles) was performed. Survival probabilities were estimated using the Kaplan-Meier method, and differences between groups were evaluated with the log-rank test. All statistical analyses were conducted using R software (version 4.2.0), with a two-sided p-value <0.05 considered statistically significant.

**Comment 15: The presentation of the exhibits can be improved: what is the unit of Time (Fig. 2). Particapants (typo), twice (Fig S1). HR (may be obvious to many readers) still needs definition in the footnote (Table 2).**

**Response 15:**

We sincerely appreciate the reviewer's careful evaluation and constructive feedback, which has helped improve the clarity and accuracy of our manuscript.

In response to the reviewer's comments, we have made the following revisions: Time unit in Figure 2: The X-axis label has been updated to explicitly indicate "Time (months)."

Typographical error: "Particapants" has been corrected to "Participants" throughout the manuscript. Definition of HR: A footnote has been added to Table 2 specifying "HR, hazard ratio.

***Figure.S1*** *The flow chart of the study (Revised)*

**Comment 16:** We've checked your submission and before we can proceed, we need you to address the following issues:1. We note your data availability statement:

"The datasets used in this study are publicly accessible online. The repository name and accession numbers can be found at https://www.cdc.gov/nchs/nhanes/index.html."

Before we proceed, please ensure that your data are publicly available at the URL provided or provide any direct URL(s), accession numbers, instructions, and additional information (DOI, data set title, etc.) required for researchers to access the specific data underlying the results presented in your study.

**Response 16:**

Thank you for your inquiry. I have uploaded the raw data in CSV format as Supporting Information (S1). The revised statement is as follows:

*The datasets used in this study are publicly accessible online. The repository name and accession numbers can be found at https://www.cdc.gov/nchs/nhanes/index.html. The dataset used for our analysis can be found in S1 RDW_Breast Cancer data.( Original data for this study.).* *The URL for the raw data is:https://figshare.com/account/articles/29207858?file=55028801. The DOI for this dataset is:10.6084/m9.figshare.29207858.*

**Reference**

1.	Olafsson HB, Sigurdarson GA, Christopher KB, Karason S, Sigurdsson GH, Sigurdsson MI. A retrospective cohort study on the association between elevated preoperative red cell distribution width and all-cause mortality after noncardiac surgery. Br J Anaesth. 2020;124: 718–725. doi:10.1016/j.bja.2020.02.009

2.	Jie Chee Y, Seneviratna A, Joo Lim C, Chiong CX, Peh DS, Hawkins R, et al. Red cell distribution width is associated with mortality and cardiovascular complications in diabetes mellitus in singapore. Eur J Prev Cardiol. 2020;27: 216–219. doi:10.1177/2047487319836854

3.	Hong J, Hu X, Liu W, Qian X, Jiang F, Xu Z, et al. Impact of red cell distribution width and red cell distribution width/albumin ratio on all-cause mortality in patients with type 2 diabetes and foot ulcers: A retrospective cohort study. Cardiovasc Diabetol. 2022;21: 91. doi:10.1186/s12933-022-01534-4

4.	Seo YJ, Yu J, Park J-Y, Lee N, Lee J, Park JH, et al. Red cell distribution width/albumin ratio and 90-day mortality after burn surgery. Burns Trauma. 2022;10: tkab050. doi:10.1093/burnst/tkab050

5.	Bazick HS, Chang D, Mahadevappa K, Gibbons FK, Christopher KB. Red cell distribution width and all-cause mortality in critically ill patients*: Critical Care Medicine. 2011;39: 1913–1921. doi:10.1097/CCM.0b013e31821b85c6

6.	Hao M, Jiang S, Tang J, Li X, Wang S, Li Y, et al. Ratio of red blood cell distribution width to albumin level and risk of mortality. JAMA Netw Open. 2024;7: e2413213. doi:10.1001/jamanetworkopen.2024.13213

7.	Fu W, Hu F, Xu C. Association between red blood cell distribution width/albumin ratio and all-cause mortality or cardiovascular diseases mortality in patients with diabetic retinopathy: A cohort study. PLoS One. 2023;18: e0296019. doi:10.1371/journal.pone.0296019

8.	Xiao J, Tan L, Pei Y, Yang R, Li J, Feng Y, et al. Association between red cell distribution width and all-cause mortality in patients with breast cancer: a retrospective analysis using MIMIC-IV 2.0. PLoS One. 2024;19: e0302414. doi:10.1371/journal.pone.0302414

9.	Cai Y, Zhaoxiong Y, Zhu W, Wang H. Association between sleep duration, depression and breast cancer in the united states: a national health and nutrition examination survey analysis 2009–2018. Ann Med. 56: 2314235. doi:10.1080/07853890.2024.2314235

10.	Ganji V, Sukik L, Hoque B, Boutefnouchet L, Shi Z. Association of Serum 25-Hydroxyvitamin D Concentration with Breast Cancer Risk in Postmenopausal Women in the US. J Pers Med. 2022;12: 944. doi:10.3390/jpm12060944

11.	Dai L, Li R, Hao Q, Bao Y, Hu L, Zhang Y, et al. Breast cancer is associated with coronary heart disease: a cross-sectional survey of NHANES 1999–2018. Front Cardiovasc Med. 2023;10: 1274976. doi:10.3389/fcvm.2023.1274976

12.	Rondanelli M, Perna S, Alalwan TA, Cazzola R, Gasparri C, Infantino V, et al. A structural equation model to assess the pathways of body adiposity and inflammation status on dysmetabolic biomarkers via red cell distribution width and mean corpuscular volume: A cross-sectional study in overweight and obese subjects. Lipids Health Dis. 2020;19: 154. doi:10.1186/s12944-020-01308-5

13.	Jain V, Nath P, Patro S. Evaluation of diagnostic accuracy of inflammation markers [neutrophil-lymphocyte ratio (NLR), red cell distribution width (RDW), prognostic nutritional index (PNI) and lymphocyte monocyte ratio (LMR)] for outcomes in patients with acute pancreatitis. J Assoc Physicians India. 2022;70: 11–12.

14.	Katamreddy A, Kokkinidis DG, Miles JA, Faillace RT. Elevated red cell distribution width is associated with negative P wave amplitude in lead v1: National health and nutrition examination survey (NHANES III). Am J Cardiovasc Dis. 2020;10: 356–361.

15.	Danese E, Lippi G, Montagnana M. Red blood cell distribution width and cardiovascular diseases. J Thorac Dis. 2015;7: E402–E411. doi:10.3978/j.issn.2072-1439.2015.10.04

16.	Arkew M, Gemechu K, Haile K, Asmerom H. Red blood cell distribution width as novel biomarker in cardiovascular diseases: a literature review. J Blood Med. 2022;13: 413–424. doi:10.2147/JBM.S367660

17.	Spadaro S, Taccone FS, Fogagnolo A, Franchi F, Scolletta S, Ragazzi R, et al. The effects of blood transfusion on red blood cell distribution width in critically ill patients: a pilot study. Transfusion. 2018;58: 1863–1869. doi:10.1111/trf.14759
